# Agranulocytosis and hepatic toxicity with ticlopidine therapy: a case report

**DOI:** 10.1186/1752-1947-4-269

**Published:** 2010-08-12

**Authors:** Antonino M Previtera, Rossella Pagani

**Affiliations:** 1 University of Milan, Department of Medicine, Surgery and Dentistry, Rehabilitation Unit, San Paolo Hospital, Milan, Italy; 2 Rehabilitation Unit, San Paolo Hospital, Milan, Italy

## Abstract

**Introduction:**

Ticlopidine is a platelet inhibitor used to prevent thrombosis in patients with cerebrovascular or coronary artery disease. The most common side effects are mild and transitory: diarrhea, dyspepsia, nausea and rashes. More serious, but less frequent, adverse effects are hematological dyscrasia and cholestatic hepatitis. We report a rare case of agranulocytosis associated with hepatic toxicity, probably related to the use of ticlopidine.

**Case presentation:**

A 70-year-old Caucasian woman, with no previous history of hematological or liver diseases, was treated with ticlopidine 250 mg twice daily immediately after a vertebrobasilar stroke. Upon admission, her blood tests were normal. About four weeks later she developed agranulocytosis and hepatic toxicity. Ticlopidine was discontinued immediately, and aspirin 25 mg and dipyridamole 200 mg were given twice daily. She was treated with hematopoietic growth factors (granulocyte colony stimulating factor), with a rapidly increased white blood count and progressive normalization of liver tests as a result.

**Conclusion:**

In the first three months following initiation of ticlopidine therapy, regular monitoring of complete blood cell count and of liver function tests is essential for the early detection of serious and unpredictable side effects.

## Introduction

Ticlopidine is a thienopyridine derivative with platelet inhibitor capability. It acts by inhibiting the platelet aggregation induced by adenosine diphosphate and by blocking the membrane receptors of fibrinogen. It is used to prevent thrombosis in patients with cerebrovascular or coronary artery disease. Two randomized clinical studies [1,2] proved the drug's efficacy versus placebo [1] and aspirin [2] in reducing the risk of transient ischemic attack and stroke in patients with a history of cerebrovascular events. Because of its adverse effects, the use of this drug is reserved for patients in whom aspirin is contraindicated, not tolerated, or when treatment with aspirin fails.

The most common side effects are mild and transitory: diarrhea, dyspepsia, nausea and rashes. More serious, but less frequent, adverse effects are hematological dyscrasia (particularly agranulocytosis, aplastic anemia, neutropenia, pancytopenia, thrombocytopenia and thrombotic thrombocytopenia purpura) and cholestatic hepatitis. However, to our knowledge, there are only a few published reports of the simultaneous occurrence of hematological and hepatic toxicity induced by ticlopidine. We report a case of agranulocytosis associated with cholestatic hepatitis related to the use of ticlopidine.

## Case presentation

A 70-year-old Caucasian woman was admitted to our Rehabilitation Ward (San Paolo Hospital, Milan) because of gait ataxia after right bulbar stroke, which occurred 10 days previously. Her medical history pointed out hypertension and hypercholesterolemia. She had no prior history with regard to hematological or liver diseases, alcohol abuse or blood transfusion. Her habitual medications were aspirin 100 mg/day, atorvastatin 20 mg/day and amlodipine 5 mg/day. Immediately after her stroke, she discontinued aspirin and started therapy with ticlopidine 250 mg twice daily.

Upon admission, her blood tests were normal. About four weeks later, she developed agranulocytosis. Her white blood count was 2600 cells/μL (reference range: 4000 to 10,000 cells/μL), neutrophil count was 100 cells/μL (reference range: 2000 to 7000 cells/μL), and liver function tests revealed a mixed cholestasis and hepatocellular injury. She had no fever and she was asymptomatic. She had elevated levels of alanine aminotransferase (560 U/L, reference range 5 to 41 U/L), of aspartate aminotransferase (551 U/L, reference range 5 to 41 U/L), of γ-glutamyl transpeptidase (449 U/L, reference range 11 to 50 U/L), of alkaline phosphatase (821 U/L, reference range 98 to 279 U/L). Total and direct bilirubin and the coagulation tests were normal.

Serology tests for hepatitis A, B and C, and for Epstein-Barr virus (EBV) and cytomegalovirus (CMV) were negative. The anti-nuclear antibodies (ANA), the anti-mitochondrial antibodies (AMA) and anti-smooth muscle antibodies (LKM) were all negative. Cobalamin and folate dosages were normal.

An abdominal ultrasound scan showed liver steatosis but did not highlight any alterations in the intra-hepatic and extra-hepatic biliary pathways and, in particular, no sign of dilatation emerged. A bone marrow aspirate showed myeloid maturation arrest, with decreased myeloid precursors and immature forms, like by iatrogenic attack. A cytogenetic analysis on bone marrow blood was 46, XX.

Ticlopidine was immediately discontinued. Aspirin 25 mg and dipyridamole 200 mg twice daily were started [3]. She was treated with granulocyte colony stimulating factor (Filgastrim, 0.3 mg/day) with an excellent evolution. On the second day, her white blood count was normal (white blood cells: 5300 cells/μL; neutrophil count 1500 cells/μL); her liver function tests progressively got better with normalization after four weeks. A liver biopsy was not performed because of her serious hemathological dyscrasia and the self-limiting nature of liver disorder.

The pathogenesis of the various types of toxic effects associated with ticlopidine therapy is unclear. There is no test available that can confirm the diagnostic hypothesis of the drug toxicity apart from the exclusion of other possible causes and the normalization of the blood tests after the drug discontinuation.

In our patient, ticlopidine may have been responsible of concomitant hematological and hepatic toxicity. In fact, other diagnostic hypothesises were excluded and when the drug was discontinued, the blood cell count and the liver function tests rapidly normalized.

The onset of hematological dyscrasia is temporally related to the initiation of ticlopidine therapy, generally occurring within the first three months, and the dyscrasia resolves within three weeks after discontinuation of therapy [4].

The latent period between the introduction of ticlopidine and the appearance of hepatotoxicity is variable, ranging from one week to six months, but it is in the range of two to 12 weeks in most patients [5]. Hepatic toxicity is not dose dependent [6] and is not related to the treatment duration [7]. When ticlopidine is discontinued, symptoms and liver abnormalities usually resolve within one to three months. In the cases of drug-induced hepatotoxicity, the liver biopsy can suggest but not establish the diagnosis, and is mainly directed to exclude other diagnosis.

While severe neutropenia is a life-threatening adverse effect due to the occurrence of fatal infections, there are no fatal cases and no irreversible hepatic damages. The only reported fatal case was due to the co-occurrence of neutropenia, which led to septic shock [8].

We emphasize that, in the first three months following initiation of ticlopidine therapy, besides a complete blood cell count, periodic checks of liver function are recommended. Hepatic toxicity induced by ticlopidine is underestimated. Regular monitoring of complete blood cell count and of liver function tests is important for prompt detection and treatment of adverse reactions but is unlikely to prevent their occurrence altogether.

## Conclusions

In the first three months after starting ticlopidine therapy, regular monitoring of complete blood cell counts and of liver function tests should be recommended for the early detection of serious side effects, even if infrequent.

## Competing interests

The authors declare that they have no competing interests.

## Authors' contributions

AMP designed the study, treated our patient, drafted the manuscript, and contributed to the data collection. RP helped to design the study, treated our patient, contributed to manuscript drafts, and contributed to the data collection. All authors have read and approved the final version of the manuscript.

## Consent

Written informed consent was obtained from the patient for publication of this case report and any accompanying images. A copy of the written consent is available for review by the Editor-in-Chief of this journal.
